# IL-17A Promotes the Migration, Invasion and the EMT Process of Lung Cancer Accompanied by NLRP3 Activation

**DOI:** 10.1155/2022/7841279

**Published:** 2022-10-30

**Authors:** Wenping Liu, Miaomiao Xin, Qing Li, Linqian Sun, Xiao Han, Jibo Wang

**Affiliations:** Department of Rheumatology & Clinical Immunology, The Affiliated Hospital of Qingdao University, Qingdao, China

## Abstract

**Background:**

Lung cancer is a deadly cancer worldwide, and its pathogenesis and treatment methods require continuous research and exploration. As a representative factor of adaptive immunity, the role of interleukin-17A (IL-17A) in lung cancer is still unclear. The purpose of the present study was to investigate the effect of IL-17A on the biological behaviour of lung cancer cells and the relative mechanism.

**Methods:**

The human lung adenocarcinoma A549 and H1299 cell lines were used for in vitro study. The effects of IL-17A on cell proliferation, migration and invasion were assessed by CCK-8 assay, wound-healing assay, transwell invasion assay and real-time cell analysis (RTCA). The expression levels of marker proteins in the process of epithelial-mesenchymal transition (EMT) were detected by western blot analysis. Caspase-1 activity and the concentration of IL-1*β* after NLRP3 inflammasome activation were measured by a Caspase-1 Activity Assay Kit and an IL-1*β* ELISA kit, respectively.

**Results:**

Compared to the control group, IL-17A treatment did not affect the proliferation of A549 and H1299 cells in vitro, but it promoted cell migration, invasion and the EMT process. IL-17A treatment increased NLRP3 expression, caspase-1 activity and IL-1*β* level. Blockade of NLRP3 alleviated the cell migration, invasion and the EMT process induced by IL-17A.

**Conclusions:**

In conclusion, these findings indicated that NLRP3 participates in the migration, invasion and the EMT process of IL-17A-stimulated lung cells in vitro.

## 1. Introduction

Lung cancer is the most commonly diagnosed malignant carcinoma around the world [1, 2], about 85% of lung cancers are non-small cell lung cancers (NSCLCs). With the features of malignant proliferation and metastasis, NSCLC has a poor prognosis [3, 4], and it has become one of the “deadly diseases” threatening human life and health [5, 6].

The pathogenesis of lung cancer is complex [7]. As the two major risk factors for lung cancer, smoking and chronic infection play crucial roles in the occurrence and development of lung cancer [8, 9]. Thus, researchers are increasingly focusing on the relationship between chronic inflammation and cancer. Inflammation is caused by the combined forces of many immune cells. Related cytokines of inflammation have been demonstrated to promote cancer occurence, and it was found to be associated to all aspects of tumour development, including tumour proliferation, invasion and metastasis, as well as angiogenesis [10]. In addition, some cytokines such as TNF-*α*, IL-6 and IL-8 has been reported to correlate with tumour prognosis. Therapies targeting these cytokines had a certain effect on tumor control [11].

In particular, as a bridge of innate and adaptive immunity, Th cells and interleukin-17A (IL-17A) cytokine secreted after stimulation become a new focus of tumour immunity [12]. The IL-17 family comprises six members: IL-17A-F. Among them, IL-17A is the most important cytokine. The role of IL-17A in lung tumours is still controversial. Some studies have shown that IL-17A contributes to reducing tumour growth and metastasis and improves prognosis [13]. Besides, some anticancer drugs, such as PI3K pathway inhibitors and Toll-like receptor agonists, are inseparable from the role of IL-17A in their anticancer effects [14]. However, other studies suggested that IL-17A promotes cancer. The expression of IL-17A in human lung cancer specimens is positively correlated with tumour microvessel and lymphatic vessel density, and it facilitates tumour metastasis [14]. Furthermore, IL-17A induces the formation of immunosuppressive microenvironment [15]. In vitro studies have also reported that IL-17A have tumour-promoting effects in lung cancer [16]. Therefore, understanding the mechanism of IL-17A in lung cancer can provide more options for its treatment.

KEGG analysis has found that the inflammatory signalling pathway, including NF-*κ*B, were downstream of IL-17A. And the activation of NF-*κ*B can activate NLRP3 [17], thus, induces the production of a variety of inflammatory mediators, ultimately promoting the amplification of the inflammatory response. NLRP3 is the most representative type of inflammasome. NLRP3 is formed by the assembly of the NLRP3 NOD-like receptor, an adaptor protein containing a caspase recruitment domain (ASC) and pro-caspase-1. Once activated, the NLRP3 inflammasome triggers caspase-1 to an active form, which subsequently cleaves pro-IL-1*β* and pro-IL-18 to its mature bioactive forms [18]. In some inflammatory diseases, IL-17A has been found to activate inflammasomes, which in turn affects the progression of the disease [19, 21]. Previous studies have also found that NLRP3 enhances the proliferation and migration of A549 lung cancer cells [22]. However, to date, there has been no relevant research on the role of NLRP3 in lung cancer cells stimulated by IL-17A. Therefore, the aim of the present study was to investigate the effects of IL-17A on A549 and H1299 lung adenocarcinoma cells in vitro and to determine whether NLRP3 is involved in the IL-17A-mediated effects.

## 2. Materials and Methods

### 2.1. Reagents

E- cadherin (24E10), caspase-1 (D7F10), NLRP3 (D4D8T), vimentin (D21H3), GAPDH (D16H11) and cleaved caspase-1 p20 (Asp297) (D57A2) antibodies were purchased from Cell Signalling (Danvers, MA, USA). Antibody against IL-1*β* (AF5103) was purchased from Affinity Biosciences (Cincinnati, OH, USA). Antibodies against IL-17A were purchased from Servicebio (Wuhan, China). *α*-SMA antibody and HRP-conjugated goat anti-rabbit secondary antibody was purchased from Absin (Shanghai, China). Recombinant human IL-17A was acquired from MedChemExpress (Princeton, NJ, USA), and 0.1% bovine serum albumin (BSA) was reconstituted in phosphate buffered saline (PBS). NLRP3 siRNA and the negative control were synthesized by GenePharma (Shanghai, China). The sequences targeting NLRP3 used in our experiments were as follows: 5′-CAACAGGAGAGACCUUUAUTT-3′ and antisense, 5′-AUAAAGGUCUCUCCUGUUGTT-3′. For the transfection of siNLRP3, Lipofectamine 3000 reagent (Invitrogen CA, USA) was used.

### 2.2. Immunohistochemistry (IHC)

Immunohistochemistry staining was used to evaluate the expression of IL-17A in lung cancer tissue. In brief, paraffin sections of lung tissues (4 *μ*M) were incubated with rabbit antibody against IL-17A (diluted 1 : 200, Servicebio) overnight at 4°C. Then, paraffin sections were incubated with goat anti-rabbit IgG (Servicebio, Wuhan, China) for 1 h at room temperature. Thereafter, sections were stained with DAB solution (Servicebio, Wuhan, China), and cell nuclei were counterstained with haematoxylin. A light microscope Leica Qwin System (Leica, Germany) was used to photograph the results of each section. This study was performed in line with the principles of the Declaration of Helsinki. Approval was granted by the Ethics Committee of the Affiliated Hospital of Qingdao University (reference number: QYFY WZLL 27155).

### 2.3. Cell Culture and Treatment

The A549 and H1299 cell lines were obtained from Procell (Wuhan, China), these two cell lines were authenticated by short tandem repeat (STR) profiling and tested for mycoplasma contamination. A549 cells were incubated in F-12 K medium while H1299 cells were incubated in RPMI 1640 medium (Procell, Wuhan, China) with 1% penicillin/streptomycin and 10% foetal bovine serum (Gibco, USA) at 37°C with 5% CO2.

### 2.4. Cell Viability Assay

Cells were seeded in a 96-well plate at a density of 1 × 10^4^ cells/well overnight, treated with different concentrations of IL-17A (50 ng/ml and 100 ng/ml) and incubated for 24 h and 48 h at 37°C with 5% CO2. Then microlitres of Cell Counting Kit-8 (CCK-8) (MCE, Princeton, NJ, USA) solution was added to each well and incubated for 2 h in darkness at 37°C. The optical density (OD) value of the samples was determined at 450 nm with a TECAN Infinite 200 PRO series (Mannedorf, Switzerland).

### 2.5. RNA Interference

To determine the role of the NLRP3 inflammasome in IL-17A (100 ng/ml)-induced epithelial-mesenchymal transition (EMT), cells were treated with a specific small interfering RNA(siRNA) targeting NLRP3 and a negative control siRNA. In brief, cells (2 × 10^5^ per well) were seeded in a 12-well plate and transfected with 100 nM siRNA using Lipofectamine 3000 (Invitrogen, CA, USA) according to the manufacturer's instructions. RNA extraction and western blot analysis were performed to confirm the interference efficiency at 24 h and 48 h.

### 2.6. Wound-Healing Assay

Cells (3 × 10^5^ cells/well) were cultured in 60-mm tissue culture dishes until they reached 90%-100% confluence as a monolayer. The monolayer was scratched with a sterile 200-*μ*l pipette tip across the centre of each dish. After scratching, the culture medium was removed, and the dishes were gently washed three times with serum-free medium to remove the detached cells. The culture dishes were then replenished with serum-free medium. Then the control group was treated with 0.1% BSA, and IL-17A treatment group was treated with 50 ng/ml and 100 ng/ml recombinant human IL-17A. In siRNA knockdown experiments, cells were firstly transfected with 100 nM control siRNA or 100 nM siRNA targeting NLRP3 using Lipofectamine 3000 transfection reagent according to the manufacturer's instructions. After 24 h, the monolayer cells were scratched and then treated with 100 ng/ml recombinant human IL-17A. Photomicrographs of the same wound position were captured at 0 h, 24 h and 48 h under an inverted microscope (×100). The migration results were evaluated by ImageJ software (National Institutes of Health, Bethesda, MD, USA). Images were acquired at the indicated time points after wound induction using an inverted microscope with a Leica QWin System (Leica, Germany). Each assay was replicated three times.

### 2.7. Invasion Assay

For the transwell chamber migration assay, a transwell system (24-well, 8 *μ*m, Costar, Corning Inc., NY, USA) was used. Cells were trypsinized and resuspended in serum-free culture medium, and the cell density was then adjusted to 1x10^4^ cells/ml. For the invasion assay, the chamber was coated with Matrigel (BD Biosciences, Franklin Lakes, NJ, USA). Next, 200 *μ*l of serum-free cell suspension medium was added to each upper chamber, and 600 *μ*l of culture medium containing 30% FBS was added to the lower chamber. After 24 h of incubation at 37°C and 5% CO2, nonmigrating cells on the top of the membrane were gently removed with a cotton swab. Migrated cells were fixed with 4% paraformaldehyde (PFA) at room temperature for 20 mins, and cells were then stained with 0.1% crystal violet (Beyotime, China) for 5 mins. The excess staining solution was then washed off with 1 × PBS. The migrated cells were imaged and counted with a phase-contrast microscope. Finally, the invasive cell numbers were counted and averaged in five random fields at 100× magnification.

### 2.8. Real Time Cell Analysis (RTCA)

Quantitative analysis of cell proliferation and migration was monitored using the xCELLigence Real-Time Cell Analysis (RTCA) [1, 2] DP instrument with an E-Plate 16 (ACEA Bioscience Inc., San Diego, CA) and a CIM-Plate 16 (ACEA Bioscience Inc., San Diego, CA) at 37°C with 5% CO2 according to the instructions. For cell proliferation, cells were seeded on E-plates at a density of 5000 cells/well with an additional 100 *μ*l of culture medium containing 10% serum. IL-17A (50 ng/ml or 100 ng/ml) was added to the treatment group. The program was set to record impedance every 15 minutes.

For migration, electrical impedance changes were measured using a gold microelectrode plated on the bottom of a membrane separating the upper and lower chambers. The lower compartment was supplemented with 10% FBS-containing medium, and 5000 cells in 100 *μ*l of serum-free medium were added to the upper compartment of the CIM plate. The cell impedance properties produced as the cells attached and detached from the gold electrodes in the CIM were monitored and recorded every 15 min. In the siRNA group, cells were harvested at 48 h after infection with NLRP3-siRNA, diluted and seeded into 16-well CIM plates in the upper chambers at a density of 5000 cells/well. The xCELLigence system automatically monitors the impedance value of each well for 24 hours.

### 2.9. RNA Extraction and Real Time-Quantitative Polymerase Chain Reaction (RT-qPCR)

Total RNA was extracted using TRIzol reagent (TaKaRa, Dalian, China) according to the manufacturer's instructions. Total RNA was reverse-transcribed to cDNA with a reverse transcription kit (Vazyme, Nanjing, China). Quantitative real-time PCR was performed using SYBR green (Vazyme, Nanjing, China) on a LightCycler® 96 System (Roche Diagnostics International), and the mRNA levels were normalized to GAPDH. Relative gene expression (fold change) was analysed using the 2^−*ΔΔ*Ct^ method according to a previous study. All primer sequences used in the present study are shown in Table 1.

### 2.10. Western Blotting

Ice-cold RIPA buffer (Elabscience, China), containing protease inhibitor (Elabscience, China), was used to extract cell protein, and the protein concentration was detected by the bicinchonininc acid (BCA) method. Protein (30 *μ*g) was loaded into each well on a 10% or 12% SDS–PAGE gel for electrophoresis, and the protein was then transferred to a PVDF membrane. After blocking with 5% nonfat milk for 1 h at room temperature, the PVDF membrane was incubated with primary antibodies at 4°C overnight. After washing with TBST, the membrane was incubated with HRP-conjugated secondary antibodies in blocking buffer at room temperature for 1 h. After three 5 min washes with TBST, the protein bands were visualized using FluorChem Q (ProteinSimple, San Jose, CA, USA) and quantified by ImageJ software (National Institutes of Health, Bethesda, MD, USA). The GAPDH signal was used to calculate the relative expression of the target protein.

### 2.11. Immunofluorescence Staining

Immunofluorescence was used to detect the expression of E-cadherin and vimentin in cells. NLRP3 in cells was knocked down by si-NLRP3 before IL-17A stimulation for 24 h. Cells were then washed with 1 × PBS, fixed in 4% PFA for 20 min and then permeabilized with 1 mL of 0.5% Triton X-100 (Solarbio, China) for 15 min. The coverslips were then blocked with 5% BSA (Solarbio, China) for 30 min, and cells were incubated with anti-E-cadherin antibody ((67A4): sc-21791 (Santa Cruz Biotechnology, Dallas, USA)) or anti-vimentin mouse monoclonal antibody (dilution 1 : 80; Absin, China) overnight at 4°C. After three 5 min washes with PBS, cells were incubated with an Alexa 488-conjugated/CY3-conjugated goat anti-mouse secondary antibody (1 : 800; Elabscience, China) in the dark at room temperature for 1 h. Finally, DAPI (Beyotime, China) was used to label nuclei. Images were then acquired using a laser scanning confocal microscope (Leica TCS SP8, Leica, Germany).

### 2.12. Enzyme-Linked Immunosorbent Assays (ELISA)

The levels of IL-1*β* in cultured cell supernatants were collected and measured using a commercial ELISA kit (R&D Systems, Minneapolis, MN, USA) following the manufacturer's instructions.

### 2.13. Measurement of Caspase-1 Activity

A caspase-1 activity assay kit (Beyotime, China) was used to measure caspase-1 activity in cells according to the manufacturer's instructions. In brief, cells were lysed with ice-cold lysis buffer, and the protein concentration was detected by the Bradford method. The absorbance was measured at a wavelength of 405 nm with a TECAN Infinite 200 PRO series (Mannedorf, Switzerland).

### 2.14. Statistical Analysis

Data were collected from at least three independent experiments. All data are presented as the mean ± standard deviation (SD). The analysis of the data was performed using GraphPad Prism version 8.0 (GraphPad Software, USA) software. T-test was used to test the difference between two groups, while one-way analysis of variance (ANOVA) was used between multiple groups, followed by a Bonferroni multiple-comparisons test.. A value of P <0.05 was considered statistically significant.

## 3. Results

### 3.1. The Expression of IL-17A Is Elevated in Patients with NSCLC

**Figure 1:**
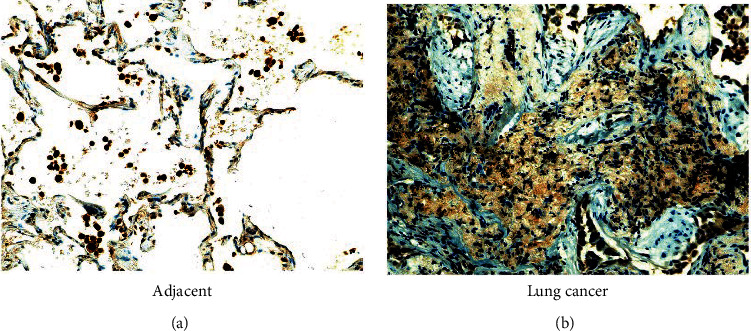
The expression of IL-17A in lung cancer tissue. (a) Representative image of IL-17A expression corresponding to adjacent normal tissues by IHC staining. (b) Representative image of IL-17A expression in NSCLC tissues by IHC staining (n =6). Magnification, ×200.

First, we detected the expression of IL-17A in lung cancer tissues and adjacent tissues. It can be found that in lung cancer tissues, the expression of IL-17A is significantly higher than that in adjacent tissues (Figures 1(a) and 1(b)).

### 3.2. IL-17A Does Not Affect the Proliferation of Lung Cells *In Vitro*

**Figure 2:**
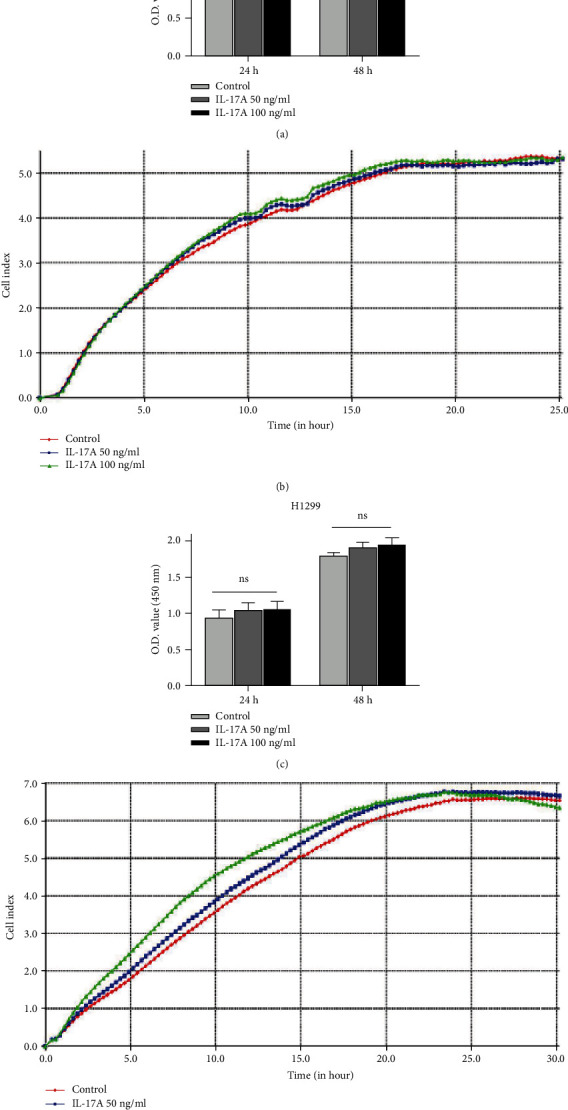
IL-17A did not affect lung cancer cells proliferation. (a) CCK-8 assay showed that IL-17A administration (50 ng/ml, 100 ng/ml) did not affect A549 cells proliferation at 24 h or 48 h compared to control group. (b) Representative real-time traces of cell index indicated that IL-17A (50 ng/ml, 100 ng/ml) treatment have a similar proliferation curve in A549 cells. (c) CCK-8 assay showed that IL-17A administration (50 ng/ml, 100 ng/ml) did not affect H1299 cells proliferation at 24 h or 48 h. (d) Representative real-time traces of cell index in each group indicated that cell proliferation does not appear to be affected by IL-17A (50 ng/ml, 100 ng/ml) treatment in H1299 cells. One-way ANOVA. Error bars, mean ± SD. ns, non-signifcant; SD, standard deviation.

To study the effects of IL-17A on lung cell proliferation, we performed CCK-8 and RTCA assays. The CCK-8 results demonstrated that IL-17A treatment did not affect A549 cell proliferation at 24 h or 48 h compared to untreated cells (Figure 2(a)) regardless of the concentration of IL-17A. Similar results were obtained by the RTCA (Figure 2(b)). And in H1299 cells, cell proliferation did not appear to be affected by IL-17A (Figures 2(c) and 2(d)). This phenomenon existed even after increasing the concentration of IL-17A (Figures 2(a)–2(d)).

### 3.3. IL-17A Promotes Migration and Invasion *In Vitro*

**Figure 3:**
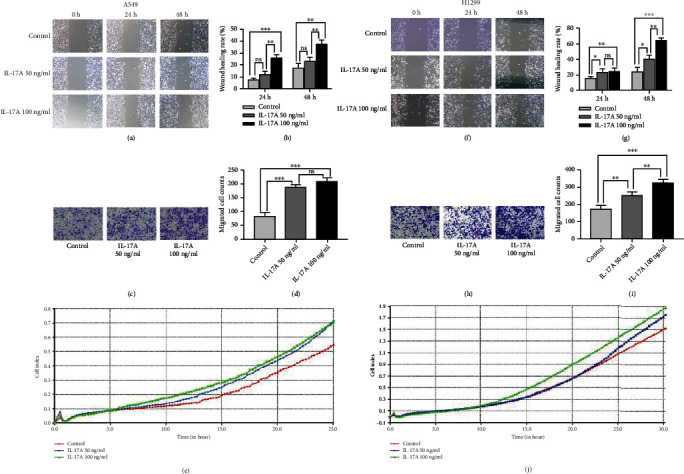
IL-17A facilitated the migration and invasion in lung cancer cells. (a) Wound healing assay showed that IL-17A administration accelerate wound healing of A549 cells. (b) Wound healing rate was calculated as (wound width of time zero–that of each time point) ÷ wound width of time zero ×100%. (c) Transwell invasion assay showed that IL-17A promotes invasion of A549 cells. Magnification, ×100. (d) Number of the cells invaded through the membrane. (e) Representative real-time traces of cell index showed that IL-17A administration promotes A549 migration. (f) Wound healing assay showed that IL-17A administration accelerate wound healing of H1299 cells. (g) Wound healing rate was calculated as (wound width of time zero–that of each time point) ÷ wound width of time zero ×100%. (h) Transwell invasion assay showed that IL-17A promotes invasion of H1299 cells. (i) Number of the cells invaded through the membrane. (j) Representative real-time traces of cell index showed that IL-17A administration promotes A549 migration. One-way ANOVA. Error bars, mean ± SD. ^∗^P <0.05; ^∗∗^P <0.01; ^∗∗∗^P <0.001, ns, non-signifcant; SD, standard deviation.

Cell migration and invasion are characteristics of tumour malignancy. We performed wound-healing assays and transwell experiments to determine whether IL-17A affects cell migration and invasion, respectively. As shown in Figures 3(a), 3(b), 3(f) and 3(g), IL-17A stimulation significantly accelerated wound healing in lung cancer cells compared to the control group. And, there were more migrated cells in the IL-17A group than that in the control group (Figures 3(c), 3(d), 3(h) and 3(i)). RTCA was then used to detect cell migration in real time (Figures 3(e) and 3(j)). And it was found that IL-17A stimulation can promote the invasion and migration of lung cancer cells *in vitro*.

### 3.4. IL-17A Promotes the EMT Process in Lung Cancer Cells

**Figure 4:**
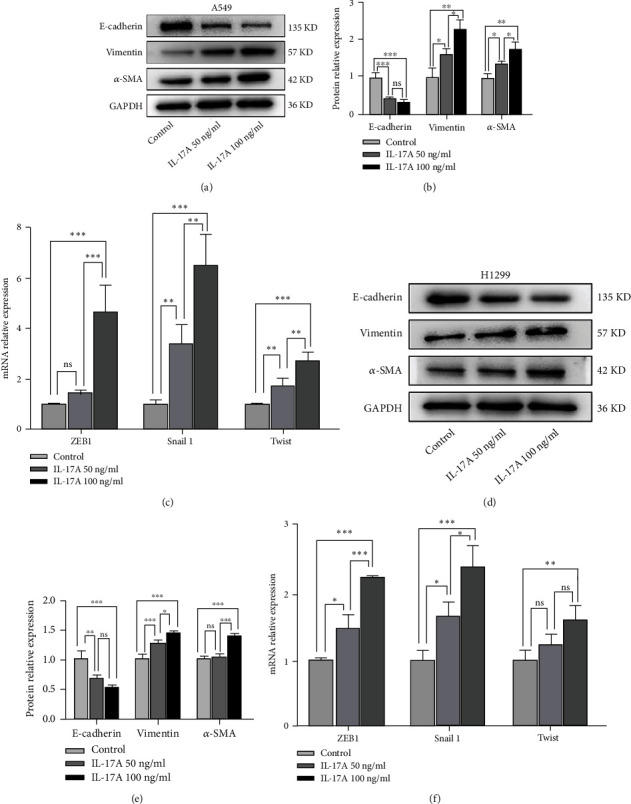
IL-17A induced the EMT process in lung cancer cells. (a) Western blot of several EMT-related markers indicated that decreased E-cadherin level while increased vimentin and *α*-SMA level after IL-17A stimulated in A549 cells. (b) Quantification of results from A. (c) The EMT-related transcription factors showed that the expression of ZEB1, Snail 1, Twist increased after IL-17A treatment in A549 cells. (d) Western blot of several EMT-related markers in each group in H1299 cells. (e) Quantification of results from d. (f) The EMT-related transcription factors in each group of H1299 cells. It is one-way ANOVA was used in statistical analysis. Error bars, mean ± SD. ^∗^P <0.05; ^∗∗^P <0.01; ^∗∗∗^P <0.001, ns, non-signifcant; EMT, epithelial-mesenchymal transition. SD, standard deviation.

The EMT process is closely related to tumour cell migration and invasion. Therefore, we also performed western blot analysis to evaluate the expression changes of the EMT-related markers after IL-17A stimulation. The expression of the epithelial cell marker, E-cadherin, was significantly downregulated with increasing IL-17A concentration compared to the blank control, while the expression of the mesenchymal cell markers, vimentin and *α*-smooth muscle actin (*α*-SMA), was significantly upregulated (Figures 4(a), 4(b), 4(d) and 4(e)). We also performed PCR analysis to measure the mRNA levels of the EMT transcription-related factors, and we found that after IL-17A stimulation, the expression of the transcription factors, ZEB1, Snail 1 and Twist, increased significantly (Figures 4(c) and 4(f)). Together, these findings demonstrated that IL-I7A promotes the EMT process in lung cancer cells in a dose-dependent manner *in vitro*. Based on these results and previous literature, a dose of 100 ng/mL IL-17A was used for further study.

### 3.5. NLRP3 Is Activated in IL-17A-Treated Lung Cancer Cells In *Vitro*

**Figure 5:**
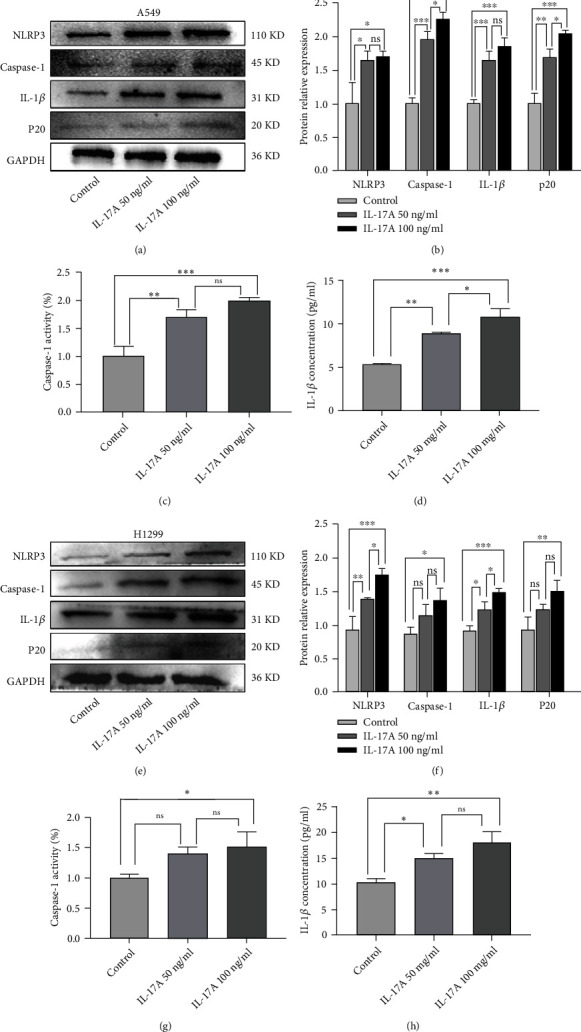
NLRP3 was activated in IL-17A treated lung cancer cells. (a) Western blot of NLRP3-related markers were increased in A549 followed by IL-17A stimulated. (b) Quantification of results from A. (c) Caspase-1 activity was increased in IL-17A group of A549. (d) The concentration of IL-1*β* in A549 was increased after IL-17A treated. (e) The expression of NLRP3-related markers in each group of H1299 cells. (f) Quantification of results from A. (g) Caspase-1 activity was increased after IL-17A stimulation. (h) IL-1*β* concentration in H1299 was increased after IL-17A treated. Data shown are mean ± SD. It is one way ANOVA was used in statistical analysis. Error bars, mean ± SD. ^∗^P <0.05; ^∗∗^P <0.01; ^∗∗∗^P <0.001, ns, non-signifcant; SD, standard deviation.

A previous study has found that NLRP3 is involved in the process of the EMT, and the activation of NLRP3 has been found in many other inflammatory diseases involving IL-17A. Therefore, we hypothesized that NLRP3 may also be activated during the EMT process induced by IL-17A. We detected the expression level of NLRP3 after cells were treated with different concentration of IL-17A. As expected, the expression of NLRP3-related markers gradually increased as the EMT process progressed after IL-17A treatment. (Figures 5(a), 5(b), 5(e), and 5(f)). In addition, the caspase-1 activity increased after IL-17A treatment (Figures 5(c) and 5(g)), and the concentration of IL-1*β* in the cell supernatant was also higher than that of the control group (Figures 5(d) and 5(h)). Therefore, NLRP3 was actived after IL-17A administration.

### 3.6. NLRP3 siRNA Inhibits IL-17A-Induced Migration and Invasion

**Figure 6:**
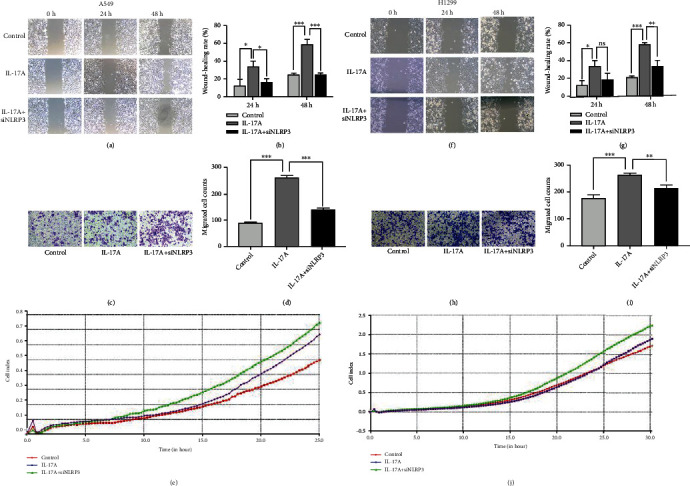
NLRP3 siRNA inhibited IL-17A induced migration and invasion. (a) Wound-healing assay showing that NLRP3 knockdown prevent migration induced by IL-17A in A549 cells. (b) Wound healing rate was calculated as (wound width of time zero–that of each time point) ÷ wound width of time zero ×100%; (c) NLRP3 siRNA impaired invasion A549 cells through Matrigel. Magnification, ×100. (d) Number of the cells invaded through the membrane. (e) Representative real-time traces of CI showed that siNLRP3 inhibit IL-17A induced migration in A549 cells. (f) Wound-healing assay showing that NLRP3 knockdown prevent migration induced by IL-17A in H1299 cells. (g) Wound healing rate in each group of H1299 cells. (h) NLRP3 siRNA impaired invasion H1299 cells through Matrigel. Magnification, ×100. (i) Number of the cells of H1299 invaded through the membrane in each group. (j) Representative real-time traces of CI showed that siNLRP3 inhibit IL-17A induced migration in H1299 cells. Student's t-test, mean ± SD are given, ^∗^P <0.05; ^∗∗^P <0.01; ^∗∗∗^P <0.001, ns, non-signifcant. CI, cell index.

To determine whether NLRP3 plays a role in IL-17A-induced migration and invasion, we transfected cells with a siRNA targeting NLRP3 and performed the above experiments. First, western blot and qPCR analysis were used to detect the protein and mRNA levels of NLRP3 after transfection of a siRNA targeting NLRP3, respectively (Supplement Figure 1a-1c, d-f). Subsequently, wound-healing assays, transwell invasion assays and RTCA were performed. We found that NLRP3 siRNA transfection significantly inhibited IL-17A-induced migration (Figures 6(a), 6(b), 6(e), 6(g), and 6(j)) and invasion (Figures 6(c), 6(d), 6(h), and 6(i)) *in vitro*.

### 3.7. NLRP3 siRNA Inhibits IL-17A-Induced the EMT Process

**Figure 7:**
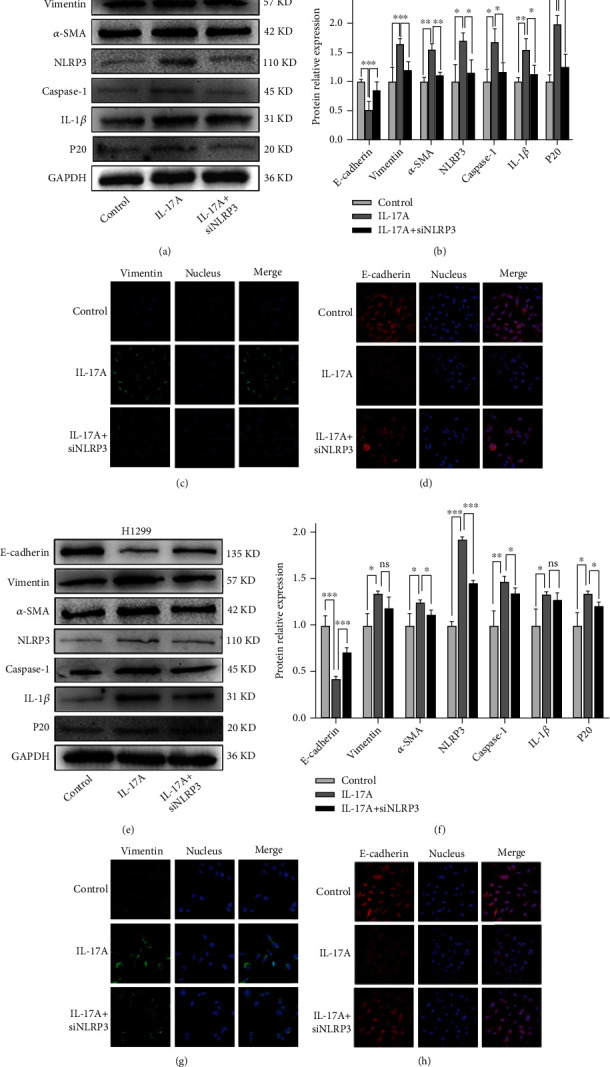
NLRP3 knockdown prevented IL-17A induced the EMT process. (a) Western blot of several EMT related markers indicated that NLRP3 siRNA increase E-cadherin level while decrease vimentin and *α*-SMA level induced by IL-17A administration in A549 cells. (b) Quantification of results from A. (c) Immunofluorescence staning of vimentin in A549 cells in each group. Magnification, ×400. (d) Immunofluorescence staning of E-cadherin in A549 cells in each group. Magnification, ×400. (e) The expression of EMT and NLRP3-related markers in each group of H1299. (f) Quantification of results from e. (g) Immunofluorescence staning of vimentin in H1299 cells in each group. Magnification, ×400. (h) Immunofluorescence staning of E-cadherin in H1299 cells in each group. Magnification, ×400. Student's t-test, mean ± SD are given, ^∗^P <0.05; ^∗∗^P <0.01; ^∗∗∗^P <0.001, ns, non-signifcant. EMT, epithelial-mesenchymal transition.

Furthermore, we performed western blot to detect the expression of EMT and inflammatory activation markers after transfection of siRNA targeting NLRP3, and we found that the expression levels of the mesenchymal cell markers, vimentin and *α*-SMA, were significantly decreased, while the epithelial cell marker, E-cadherin, was increased in the IL-17A + siRNA group compared to the IL-17A group (Figures 7(a), 7(b), 7(e) and 7(f)), further demonstrating that NLRP3 is involved in IL-17A-induced the EMT process. Subsequently, the location and expression of the mesenchymal cell marker (vimentin) and epithelial cell marker (E-cadherin) were assessed by immunofluorescence (IF) stain. Vimentin was located in the cytoplasm and E-cadherin was marked on the cell membrane. The changing trend in the expression of these two proteins were consistanted with the western blot results (Figures 7(c), 7(d), 7(g) and 7(h)).

## 4. Discussion

The effect of IL-17A on lung cancer is not yet fully understood. In this study, we studied the effect of IL-17A on the biological behavior of lung adenocarcinoma cell lines of A549 and H1299 in vitro. In general, our results were consistent with previous results [16]. Our results also found that IL-17A does not promote the proliferation of A549 cells in vitro, but it can indeed promote the migration and invasion, and promote the process of EMT. More importantly, our study found that the phenotypic changes that occurred after IL-17A stimulated A549 were accompanied by the activation of NLRP3.

There have been several relevant studies on the effect of IL-17A on lung cancer, but what changes occur in tumor cells after IL-17A stimulation and the mechanism of the changes remain to be elucidated. Ferreira et al. used IL-17A/IL-17F to stimulate A549, respectively, in their research, and they did not find that IL-17A or IL-17F showed any effect on cell proliferation and glucose metabolism [22]. Of course, this may be related to the dose of IL-17A they use. After all, more literature records that the dose of IL-17A is 100 ng/ml instead of 50 ng/ml. Nevertheless, when we stimulated A549 and H1299 cells with IL-17A of 100 ng/ml, and performed CCK8 assay and RTCA test, respectively, we also failed finding any effect on cell proliferation. Similarly, Ran and his colleagues applied IL-17A of 1000 ng/ml to stimulation A549 but failed to find any changes in cell viability [23]. Various signs indicate that IL-17A may not affect cell proliferation. The differences were that when we stimulated the cells with IL-17A of 50 ng/ml, we found that the migration and invasion capabilities of cells changed immediately, the same trend were also detected in the results of RTCA test. The migration and invasion ability of tumor can be caused by the EMT of tumor cells [24]. The EMT process refers to the loss of epithelial markers and the acquisition of mesenchymal markers. The occurrence of the EMT is one of the basic links of tumor metastasis [25]. Previously, the EMT process has been found in A549 cells after stimulated by IL-17A [26]. Our findings are consistent with before, the evidence of the EMT process was also detected in our study. This shows that IL-17A does play a role in promoting the malignant transformation of migration, invasion and the EMT process *in vitro*.

Previously, Li et al. conducted KEGG analysis on the IL-17 signaling pathway and they found that NF-*κ*B pathway participates in the downstream of IL-17. It is generally known that activation of the NF-*κ*B signaling pathway can further activate the NLRP3 inflammasome [20]. Thus, Therefore, their subsequent studies found that IL-17A can activate NLRP3, which leads to pyroptosis in pneumonia-induced sepsis. Cho *et al*. found that IL-17A treatment can enhance skin inflammation by activating NLRP3, thus to stimulate keratinocytes to secrete IL-1*β* [21]. Similarly, Zhang's research found that IL-17A can activate NLRP3 in retinal epithelial cells [27], Yan *et al.* found that IL-17A can activate NLRP3 to participate in podocyte damage [28]. What's more, NLRP3 was found to be related to the occurrence and development of a variety of tumors. NLRP3 can promote the development of mesothelioma [29] and is associated with lung metastasis [30]. Moreover, in lung cancer, previous studies have shown that NLRP3 can enhance the proliferation of A549 cells through IL-1*β*/ERK/CREB and IL-18/AKT/CREK signaling pathways, and promote tumor metastasis by reducing E-cadherin and increasing Snail expression [31]. Based on the above research, we speculate that lung cancer cells may be accompanied by changes in NLRP3 expression after IL-17A administration. Therefore, we subsequently tested the expression of NLRP3 after IL-17A stimulated A549 and H1299 cells. We were surprised to find that the expression of NLRP3 after IL-17A stimulation was higher than that of the control group. The activity of caspase-1 and the concentration of IL-1*β* in the cell culture supernatant also increased after IL-17A treatment. Combined with the changes in cell phenotype after IL-17A stimulation, we think that NLRP3 is involved in the process of IL-17A induced cell migration, invasion and the EMT process. In order to better prove that NLRP3 is involved in the process of IL-17A inducing lung cancer cells, we designed a small interfering RNA (siRNA) targeting NLRP3 and repeated the above experiment. It was found that after blocking NLRP3, the effects above after IL-17A stimulation was weakened, confirming that IL-17A can promote the migration, invasion and the EMT process of lung cancer cells by activating NLRP3 *in vitro*. Subsequent immunofluorescence experiments also confirmed the changes of the marker protein vimentin during the the EMT process.

The limitation of our research is that we did not carry out *in vivo* experiments, which could not well reflect the true effect of IL-17A on lung cancer *in vivo*.

## 5. Conclusions

In conclusion, we found that IL-17A promotes the migration, invasion and the EMT process of lung cancer cells *in vitro* without affecting cell proliferation, and this process was accompanied by NLRP3 activation.

## Figures and Tables

**Table 1:** Sequence of primers.

Name	Sense	Antisense
GAPDH	5'- AGGTCGGAGTCAACGGATTT -3'	5'- ATCTCGCTCCTGGAAGATGG -3'
ZEB 1	5'- CTTGAACGTCACATGACATCACATA -3'	5'- TCTTGCAGTTTGGGCATTCATA -3'
Snail 1	5'- GTCAGATGAGGACAGTGGGAAAG-3'	5'- AGACTGAAGTAGAGGAGAAGGACG-3'
Twist	5'- AGCAAGATTCAGACCCTCAAGC -3'	5'- TTGCCATCTTGGAGTCCAGCTC -3'
NLRP3	5'- GATCTTCGCTGCGATCAACA -3'	5'- GGGATTCGAAACACGTGCATTA -3'

## Data Availability

The data that support the findings of this study are available from the corresponding author upon reasonable request.

## Supplementary Materials

Supplementary Materials Supplement Figure 1. (a) Expression of NLRP3 protein in A549 was examined with Western blot after transfection of siRNA targeting NLRP3 for 48 h. (b) Quantification of results from A. (c) mRNA of NLRP3 in A549 was examined with qPCR after transfection of siNLRP3 for 24 h. (d) Expression of NLRP3 protein in H1299 was examined with Western blot after transfection of siRNA targeting NLRP3 for 48 h. (e) Quantification of results from A. (f) mRNA of NLRP3 in H1299 was examined with qPCR after transfection of siNLRP3 for 24 h.
