# Supplementary material for: IL-17A Promotes the Migration, Invasion and the EMT Process of Lung Cancer Accompanied by NLRP3 Activation
Source: Biomed Res Int. 2022 Oct 30;2022:7841279. doi: 10.1155/2022/7841279 (PMC9637470; doi:10.1155/2022/7841279)
Supplement: Supplementary Materials — Supplement Figure 1. (a) Expression of NLRP3 protein in A549 was examined with Western blot after transfection of siRNA targeting NLRP3 for 48 h. (b) Quantification of results from A. (c) mRNA of NLRP3 in A549 was examined with qPCR after transfection of siNLRP3 for 24 h. (d) Expression of NLRP3 protein in H1299 was examined with Western blot after transfection of siRNA targeting NLRP3 for 48 h. (e) Quantification of results from A. (f) mRNA of NLRP3 in H1299 was examined with qPCR after transfection of siNLRP3 for 24 h. [file 7841279.f1.docx]

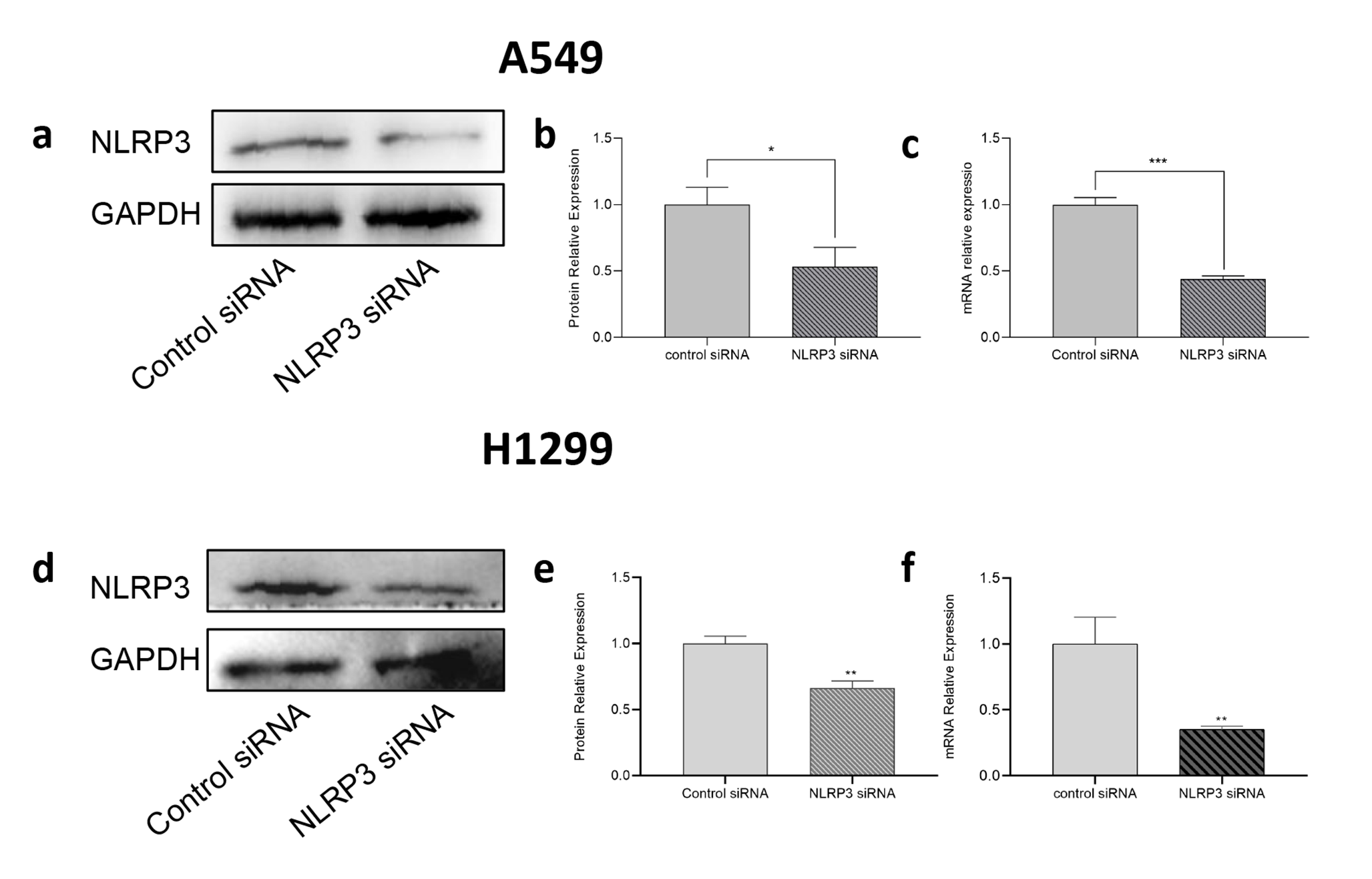


**Supplement Figure 1. (a)** Expression of NLRP3 protein in A549 was examined with Western blot after transfection of siRNA targeting NLRP3 for 48h. **(b)** Quantification of results from A. **(c)** mRNA of NLRP3 in A549 was examined with qPCR after transfection of siNLRP3 for 24h. **(d)** Expression of NLRP3 protein in H1299 was examined with Western blot after transfection of siRNA targeting NLRP3 for 48h. **(e)** Quantification of results from A. **(f)** mRNA of NLRP3 in H1299 was examined with qPCR after transfection of siNLRP3 for 24h.
